# Genome-wide identification of copy number variations between two chicken lines that differ in genetic resistance to Marek’s disease

**DOI:** 10.1186/s12864-015-2080-5

**Published:** 2015-10-23

**Authors:** Yiyuan Yan, Ning Yang, Hans H. Cheng, Jiuzhou Song, Lujiang Qu

**Affiliations:** Department of Animal Genetics and Breeding, College of Animal Science, China Agricultural University, Beijing, 100193 China; USDA, ARS, Avian Disease and Oncology Laboratory, East Lansing, MI 48823 USA; Department of Animal and Avian Sciences, University of Maryland, College Park, MD 20742 USA

**Keywords:** Copy number variation, Chicken, Susceptibility, Marek’s disease, MAPK signaling pathway, Next generation sequencing

## Abstract

**Background:**

Copy number variation (CNV) is a major source of genome polymorphism that directly contributes to phenotypic variation such as resistance to infectious diseases. Lines 6_3_ and 7_2_ are two highly inbred experimental chicken lines that differ greatly in susceptibility to Marek’s disease (MD), and have been used extensively in efforts to identify the genetic and molecular basis for genetic resistance to MD. Using next generation sequencing, we present a genome-wide assessment of CNVs that are potentially associated with genetic resistance to MD.

**Methods:**

Three chickens randomly selected from each line were sequenced to an average depth of 20×. Two popular software, CNVnator and Pindel, were used to call genomic CNVs separately. The results were combined to obtain a union set of genomic CNVs in the two chicken lines.

**Results:**

A total of 5,680 CNV regions (CNVRs) were identified after merging the two datasets, of which 1,546 and 1,866 were specific to the MD resistant or susceptible line, respectively. Over half of the line-specific CNVRs were shared by 2 or more chickens, reflecting the reduced diversity in both inbred lines. The CNVRs fixed in the susceptible lines were significantly enriched in genes involved in MAPK signaling pathway. We also found 67 CNVRs overlapping with 62 genes previously shown to be strong candidates of the underlying genes responsible for the susceptibility to MD.

**Conclusions:**

Our findings provide new insights into the genetic architecture of the two chicken lines and additional evidence that MAPK signaling pathway may play an important role in host response to MD virus infection. The rich source of line-specific CNVs is valuable for future disease-related association studies in the two chicken lines.

**Electronic supplementary material:**

The online version of this article (doi:10.1186/s12864-015-2080-5) contains supplementary material, which is available to authorized users.

## Background

Marek’s disease (MD) is a T cell lymphoma disease of chickens induced by Marek’s disease virus (MDV), an oncogenic ɑ-herpesvirus [1]. MD is characterized by lesions of visceral organs and enlarged nerves that can result in death, and continues to be one of the most serious chronic disease threats to the poultry industry. Since the early 1970s, the poultry industry has relied heavily on MD vaccines, which have greatly eliminated the incidence of MD [2, 3]. Though successful, the efficacy of vaccines has been compromised by the unpredictable outbreaks of more virulent field strains.

Improving genetic resistance to MD of chickens is a desirable and sustainable long-term MD control measure. To achieve this objective, studies have been carried out to uncover the genetic variants underlying resistance to MD. Genome-wide QTL scans have identified a number of genomic regions associated with the resistance to MD [4–7]. With modern statistical and genomic tools, Li et al. [8] reported two loci associated with MD resistance through genome-wide association study (GWAS). However, the resolution limits of these strategies make it difficult to identify the underlying causative genes, and the variants only explain a small proportion of total genetic variation [9], leaving a large part of variation unexplained [10]. More recently, Cheng et al. [11] found that SNPs in allele-specific expression (ASE) genes captures more than 83 % of the additive genetic variation of MD resistance, demonstrating that the most of the ASE genes are strong candidates of underlying genes of MD resistance. However, the causative mutations and affected pathways are still illusive.

Copy number variations (CNVs) are a type of genomic polymorphisms characterized by gains or losses of DNA copies that usually extend from 1 Kb to several million bases in length and, thus, are believed to have a great impact on phenotypes. Accumulating evidences suggest that CNVs are responsible for a number of genetic disorders and susceptibility to infectious diseases [12, 13], and probably contribute to a fraction of “missing heritability” [10, 14].

In this study, we applied deep sequencing on two experimental inbred chicken lines (Avian Disease and Oncology Laboratory line 6_3_ and 7_2_; ADOL) that differ substantially in susceptibility to MD. We hypothesized that resistance to MD is genetically controlled by some CNVs between these lines. A main focus of this study was on the detection of deletions, as this type of CNV is frequently associated with genetic disorders and infectious diseases in both human and animals [12, 15, 16]. Our analysis provides new insight of the genetic architecture of the two inbred lines and the identified CNVs are a rich resource of variation for future association studies.

## Methods

### Chicken samples

Chickens from two highly inbred chicken lines maintained in Avian Disease and Oncology Laboratory (ADOL) (line 6_3_ and line 7_2_) were used in this study. The two lines share the same major histocompatibility complex (MHC) haplotype (B2) [17–19], which is a major locus influencing MD incidence, yet differ significantly in susceptibility to MD (lines 6_3_ and 7_2_ are MD resistant and susceptible, respectively). Three chickens from each line (designated RES1, RES2, RES3 from line 6_3_ and SUS1, SUS2, SUS3 from line7_2_) were randomly selected for blood collection. The procedure of collecting blood samples of all animals were carried out followed the ADOL Animal Care and Usage Committee policy.

### Library construction and sequencing

Genomic DNAs were extracted from blood by standard phenol/chloroform method [20] and then measured for concentration and purity by NanoDrop (Thermo Fisher Scientific Inc. Waltham, MA, USA). Genomic DNAs were sheared to yield an average size of 500 bp and then ligated to Illumina (Illumina Inc., San Diego, CA, USA) paired-end adaptors. After PCR amplification and purification, the resultant DNA clusters were sequenced on an Illumina HiSeq 2000 sequencer (Illumina Inc.). Raw reads of 2 × 100 bp were generated for downstream analysis.

### Read mapping and CNV calling

Low quality reads were removed as previously described [21]. Mapping reads to the reference genome (galGal4) was performed with BWA-MEM [22], using default parameters. Removal of duplicated reads, realignment of reads around insertion and deletions were performed as previously described [21].

CNVnator (ver 0.3) [23] based on read depth (RD) method was used to predict genomic CNVs between the two chicken lines and the reference. The CNV calling pipeline employed here has been previously described [24], with slight modifications; to improve detection accuracy, only reads with quality score of 20 (Q20) or higher were used. To improve detection sensitivity, we used another software employing split-read approaches, Pindel (ver 0.2.5a4), [25] to detect medium to large structural variations (SV). The minimal mapping quality of the reads that Pindel uses as anchor was set to 20 (parameter “A”) and the maximum size of SV to be detected was set to 32,628 bp (parameter “x”). Other parameters were set to default.

To retain confident CNV calls for downstream analysis, we applied stringent filtering for raw CNV results. For CNVs called by CNVnator, only significant CNVs (*P* < 0.01) with a minimum size of 1 kb were retained. CNVs located on random contigs (chrN_random), unlocalized chromosomes (chrUn), or in overlapping gaps were discarded. For each SV predicted by Pindel, we required a minimum of 5 uniquely mapped reads supporting the variation. Similarly, variations on random contigs and unlocalized chromosomes were excluded from the analysis.

### Gene content and functional analysis

Results from CNVnator and Pindel were combined to obtain a collective set (union) of unique CNVs with different start or end coordinates. These CNVs were then merged into non-overlapping CNV regions (CNVRs) by aggregating CNVs that overlap by at least 1 bp. The Ensembl genes (release 76) overlapping with these CNVRs were extracted using custom PERL scripts. Gene ontology (GO) and Kyoto Encyclopedia of Genes and Genomes (KEGG) analysis were performed in Database for Annotation, Visualization and Integrated Discovery (DAVID, ver 6.7) [26].

### Comparison with previous CNV discovery studies and gene expression studies

Since most of previous CNV detection studies using the same two chicken lines were based on the Galgal3 genome assembly, coordinates of the CNVRs were converted using NCBI Remap (http://www.ncbi.nlm.nih.gov/genome/tools/remap). The minimum ratio of bases that must be remapped was set to 0.5, and the maximum ratio for difference between source length and the target length was set to 5.0. At the same, we also allow multiple locations to be returned and fragments to be merged. In terms of selecting the best remap results, the following criteria was applied: a) the coverage was closest to 1.0; b) the top hit was retained; and 3) results that contained “random” or “NULL” were discarded. CNVRs overlapped reciprocally at least 1 bp were considered cross-validated.

The transcripts with altered expression after MDV infection identified by ASE screening were obtained from Perumbakkam et al. [27] (Supplemental Table 7). Duplicate transcripts were removed, and then the coordinates based on Galgal4 assembly were obtained for the transcript IDs.

To access whether the overlap between fixed CNVRs and ASE genes is statistically significant, we performed permutation test using R statistical package [28]. Specifically, we generated randomly distributed CNVs of the same sizes as the tested CNVs by simulation. The number of overlap in each simulation was calculated and the empirical distribution of hits was obtained by 10,000 independent simulations. The significance of overlap was determined by setting the threshold according to the empirical distribution.

### Validation by PCR assay

In addition, PCR experiments to validate a subset of the CNV results were performed. Primers were designed by Primer Premier5 (Premier Biosoft., Palo Alto, CA, USA) [29] to amplify the entire CNVR. PCR reactions were conducted in a 20 ul volume containing 15–30 ng genomic DNA, 2–4 uM forward and backward primers, and 33–35 thermal cycles. The resultant amplicons were examined by agarose gel electrophoresis (concentration: 1.0 %).

## Availability of supporting data

The raw sequence data has been submitted to NCBI Sequence Read Achieve (SRA) under the Bioproject number of PRJNA280243. The Biosample numbers for the sequenced samples are SAMN03459116 (RES1), SAMN03438107 (RES2), SAMN03438108 (RES3), SAMN03459118 (SUS1), SAMN03459119 (SUS2), SAMN03459120 (SUS3), respectively.

## Results

### Read mapping and CNV detection

On average, ~236 million raw reads were generated for each sample, and after quality control, ~213 million reads were successfully aligned to the reference genome. The sequencing depths calculated from mapped reads were 20.5× and 20.0× for the resistant and susceptible lines, respectively (Table 1). To minimize false positives, we only used Q20 reads (effective reads, [21]) for further analysis. The average genomic coverage by Q20 reads was 95.1 %, which resulted in 19.6× and 18.8× on average coverage for the resistant and susceptible lines, respectively (Table 1).

**Table 1:** Statistics of sequencing and read mapping for each chicken

Chicken^a^	Line	Raw reads	After QC (Ratio, %)	Mapped reads (Ratio, %)	Q20 Reads (Ratio, %)	Effective depth (X)^b^	Q20 Coverage^b^ (%)
RES1	6_3_	205,596,588	187,877,511(91.3)	186,810,961(99.4)	176,987,396(94.2)	16.8	94.8
RES2	6_3_	234,886,526	207,580,222(88.4)	206,152,608(99.3)	193,530,549(93.2)	18.4	95.0
RES3	6_3_	286,301,462	256,953,147(89.8)	255,430,372(99.4)	241,909,593(94.1)	23.5	95.0
SUS1	7_2_	233,281,700	213,717,896(91.6)	212,018,941(99.2)	195,175,544(91.3)	18.6	95.4
SUS2	7_2_	222,031,616	204,055,933(91.9)	202,806,267(99.4)	190,921,220(93.6)	18.2	95.3
SUS3	7_2_	236,881,356	220,680,041(93.2)	219,476,108(99.5)	207,828,724(94.2)	19.8	94.9

^a^ *RES* resistant, *SUS* susceptible; ^b^ Calculated based on Q20 reads

A total of 57,824 CNVs were identified by CNVnator in these two lines, and 8,135 unique CNVs with different start or end coordinates passed our stringent filtering criteria (Additional file 1: Table S1). The size of these CNVs ranges from 1 to 543.5 kb, with an average of 8.4 kb. As the operational definition of a CNV become smaller in size due to the use of next generation sequencing, we also used Pindel to detect smaller structural variations. This analysis yielded 3,697 unique deletions after filtering according to our criteria, which ranged in size from 100 to 32,628 bp, with an average of 4.6 kb. On average, each chicken line harbors 3,351 CNVs. Aggregating overlapping CNVs resulted in 3,241 and 3,697 CNVRs for CNVnator and Pindel, respectively. Each of these two approaches seem to capture a portion of the whole structural variation, as about 30.9 % of CNVnator’s results and 21.6 % of Pindel’s results overlapped with the other. After combing the two datasets together, a collective set consisting of 5,680 CNVRs were obtained, which are distributed over all chromosomes and two linkage groups (LGE22C19W28_E50C23 and LGE64) (Additional file 1: Table S1). The minimum CNVR was 102 bp and the maximum was 543,600 bp, with an average of 5,096 bp and together, these CNVRs affected 29.41 Mb, which entails 2.76 % of the chicken genome. The CNVRs belonging to loss, gain, or both account for 90.4 %, 7.6 % and 2.0 %, respectively. The number of CNVRs in each chicken was 2,807 in RES1, 2,731 in RES2, 3,040 in RES3, 2,831 in SUS1, 2,928 in SUS2, and 3,079 in SUS3.

### Validation

#### Cross-validation with previous studies

About 93.7 % of Crooijmans et al.’s [30] and 44.4 % of Luo et al.’s [31] CNVs could be successfully converted to the Galgal4 assembly (Additional file 2: Table S2 and Table S3). The mean sizes of successfully converted CNV were 62.1 kb (vs. 60.4 kb before conversion) and 43.7 kb (vs. 44.9 kb before conversion), respectively. We obtained 346 and 32 CNVRs respectively for comparison. About 36.4 % of Crooijmans et al.’s [30] and 59.4 % of Luo et al.’s results [31] can be validated by our study. Taken together, 6.0 % of our CNVs overlapped with these two previous studies, and in terms of involved bases, this percentage rose to 26.1 % (Table 2; Additional file 2: Table S4).

**Table 2:** Cross-validation with previous CNV discovery studies in the same population^a^

Studies	Platform	Reference	Samples	CNVR count^b^	CNVR size (kb)				Overlap with this study			
					Total	Mean	Max	Min	CNVR #	Pct.^c^	Overlap size (kb)	Pct.^d^
This study	Next generation sequencing	Galgal4	6	5,680	29,410	5	544	0.1				
Crooijmans et al. [30]	Agilent 244 K aCGH chip	Galgal3	10	346	18,908	55	5,321	3	126	36.4	6,981	36.9
Luo et al. [31]	NimbleGen 385 k aCGH chip	Galgal3	4	32	1,399	44	190	10	19	59.4	694	49.6
Cumulated^e^	-	-	-		19,379	53	53	3	145	38.4	7,714	39.6

^a^The comparison was based on Galgal4 assembly

^b^The CNVRs were obtained by aggregating overlapping CNVs that were successfully converted to Galgal4 assembly

^c^The percentage was calculated by dividing the number of overlapped CNVRs by the total number of CNVRs in corresponding study

^d^The percentage was calculated by dividing the number of overlapped bases by the total bases affected by CNVs in corresponding study

^e^A union set based on previous studies

#### Validation by PCR assay

**Fig. 1:**
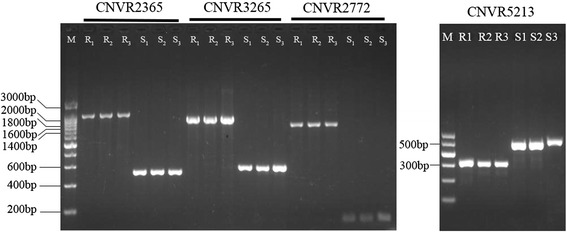
PCR validation of CNVRs. The PCR products were examined in 1 % agarose gel. M: marker (200 bp for CNVR2365,3265 and 2772; 100 bp for CNVR5213); R_1_-R_3_: the three sequenced samples in line6_3_; S_1_-S_3_: the three sequenced samples in line7_2_

We selected four CNVs (deletions, CNVR2365, CNVR2772, CNVR3265 and CNVR5213) and performed PCR validation on the sequenced chickens (primer information was provided in Additional file 3: Table S5). For most of the deletions in our studies, the CNVs are zero copies because of the highly homozygous genetic background. Therefore, the CNV status could be easily identified as presence or absence of PCR product through electrophoresis. The PCR results correspond well with the sequencing results (Fig. 1).

### CNVRs on GGA16

In chicken, GGA16 has higher interest for disease resistance as it contains the major histocompatibility complex (MHC), the key regulator of the immune system. While the two chicken lines differ significantly in resistance to MD, they share the same MHC haplotype (B2) [17, 19]. Therefore, the resistance difference between the two lines is believed to be attributed to non-MHC loci. To test this assumption, sequence variation of this chromosome was examined. Twenty CNVRs were found in this chromosome, 8 of which (CNVR4443-4450) overlapped with previous findings (Table 3). The CNVRs overlapped with several MHC genes including *BF1*, *BLEC2*, *TRIM* family (*TRIM1*, *TRIM27.2*, and *TRIM7.1*) and *BG.* No CNVs were detected in *BL* loci. It should be noted that nearly all CNVRs were found in only one chicken per line.

**Table 3:** Line-specific CNVRs on GGA16

CNVR ID	Start	End	Size (bp)	Status	Line	Sample	Overlap CNVs	Overlap genes
CNVR4435	107501	108600	1100	Loss	7_2_	SUS1	NA	ENSGALT00000000188
CNVR4437	161801	163800	2000	Loss	7_2_	SUS1	NA	ENSGALT00000044422;
								ENSGALT00000045456;
								ENSGALT00000045935;
								ENSGALT00000042650
CNVR4438	171001	172600	1600	Loss	7_2_	SUS1	NA	NA
CNVR4439	177101	179200	2100	Loss	7_2_	SUS1;SUS2	NA	ENSGALT00000000149
CNVR4441	186601	189200	2600	Loss	6_3_	RES1	NA	ENSGALT00000000149
CNVR4444	235134	251820	16687	Loss	7_2_	SUS1	Crooijmans et al. [30]	ENSGALT00000003794;
								ENSGALT00000043371
CNVR4447	402401	405200	2800	Loss	7_2_	SUS3	Crooijmans et al. [30]; Luo et al. [31]	ENSGALT00000001702
CNVR4448	408901	412800	3900	Gain	6_3_	RES1	Crooijmans et al. [30]; Luo et al. [31]	ENSGALT00000041340

### Line-specific CNVRs

**Fig. 2:**
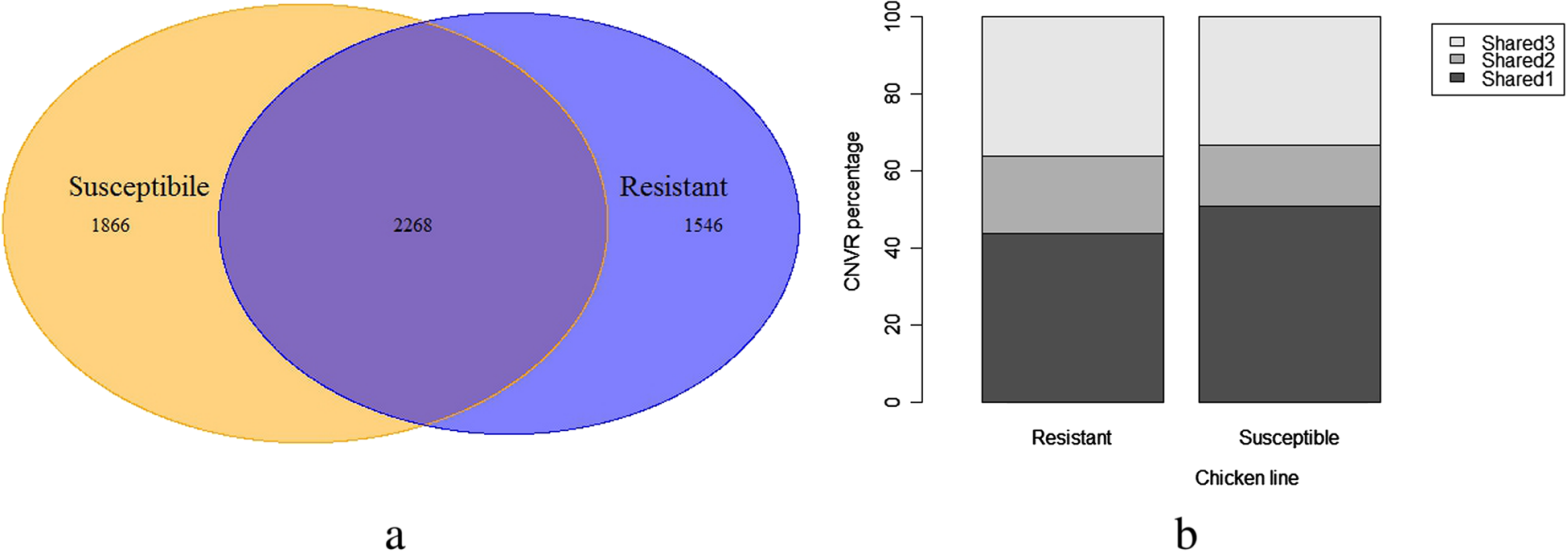
Summary of line-specific CNVRs. **a**: Line-specific and shared CNVRs in the two chicken lines. **b**: The percentage of line-specific CNVRs with different frequencies. The number after “Shared” denotes the number of chickens where this CNVR was found

Taken together, the majority of our CNVRs (71.3 %) were found in more than one chicken (Additional file 1: Table S1). And separately, about 72.2 % and 66.4 % of the CNVRs were shared by at least two individuals for resistant and susceptible lines, respectively. About 40 % of the CNVRs were shared by both lines (Fig. 2a). The CNVs unique to one line are of particular interest because they probably contribute to the unique genetic characteristics between the two lines, i.e., resistance to MD. We defined line-specific CNVRs as those found in one line while not in the other line, and obtained 1,546 (**~**3.62 Mb) and 1,866 (**~**7.03 Mb) line-specific CNVRs in the resistant and susceptible lines, respectively (Fig. 2a, Additional file 1: Table S1). Among the line-specific CNVRs, 559 and 624 CNVRs have been fixed in resistant and susceptible line, respectively. Over half of these line-specific CNVRs were shared by two or more chickens within the line (Fig. 2b). The mean CNVR size for the lines 6_3_ and 7_2_ were 2,340 bp and 3,766 bp, respectively, which was significantly different (*P* = 5.78e-10).

**Fig. 3:**
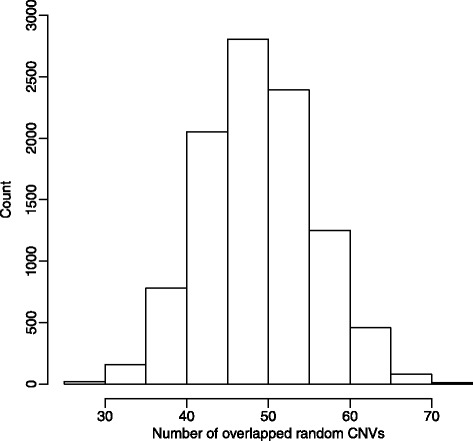
Empirical distribution of the number of overlapped random CNVs with ASE genes. Simulations are performed to test the significance of the overlap between line-specific CNVRs and ASE genes. The vertical axis shows the counts of overlap numbers in 10,000 independent simulations

Perumbakkam et al. [27] identified a number of genes showing allele-specific expression using RNA-seq. Further work by Cheng et al. [11] demonstrated that variations of these genes account for as much as 83 % of the additive genetic variation in MD resistance. Thus these genes are strong candidates of underlying genes of resistance or susceptibility to MD. To further explore the potential association of our line-specific CNVRs with MD resistance, we compared the line-specific CNVRs of high frequency (shared by two or three individuals within the line) with those ASE genes.  A total of 803 transcripts were retained for comparison (Additional file 4: Table S6). The analysis reveals that 67 (3.8 %) CNVRs overlapped with 62 (7.7 %) ASE genes (Additional file 4: Table S7). Simulation test was conducted to examine the significance of the overlap. We use 10,000 simulations to build the empirical distribution of overlaps (Fig. 3). The results showed that only 68 cases out of 10,000 independent sets exceeded the threshold. Therefore, our fixed CNVs have significant overlaps with the ASE genes (*P* = 0.0068).

### Gene content analysis

**Fig. 4:**
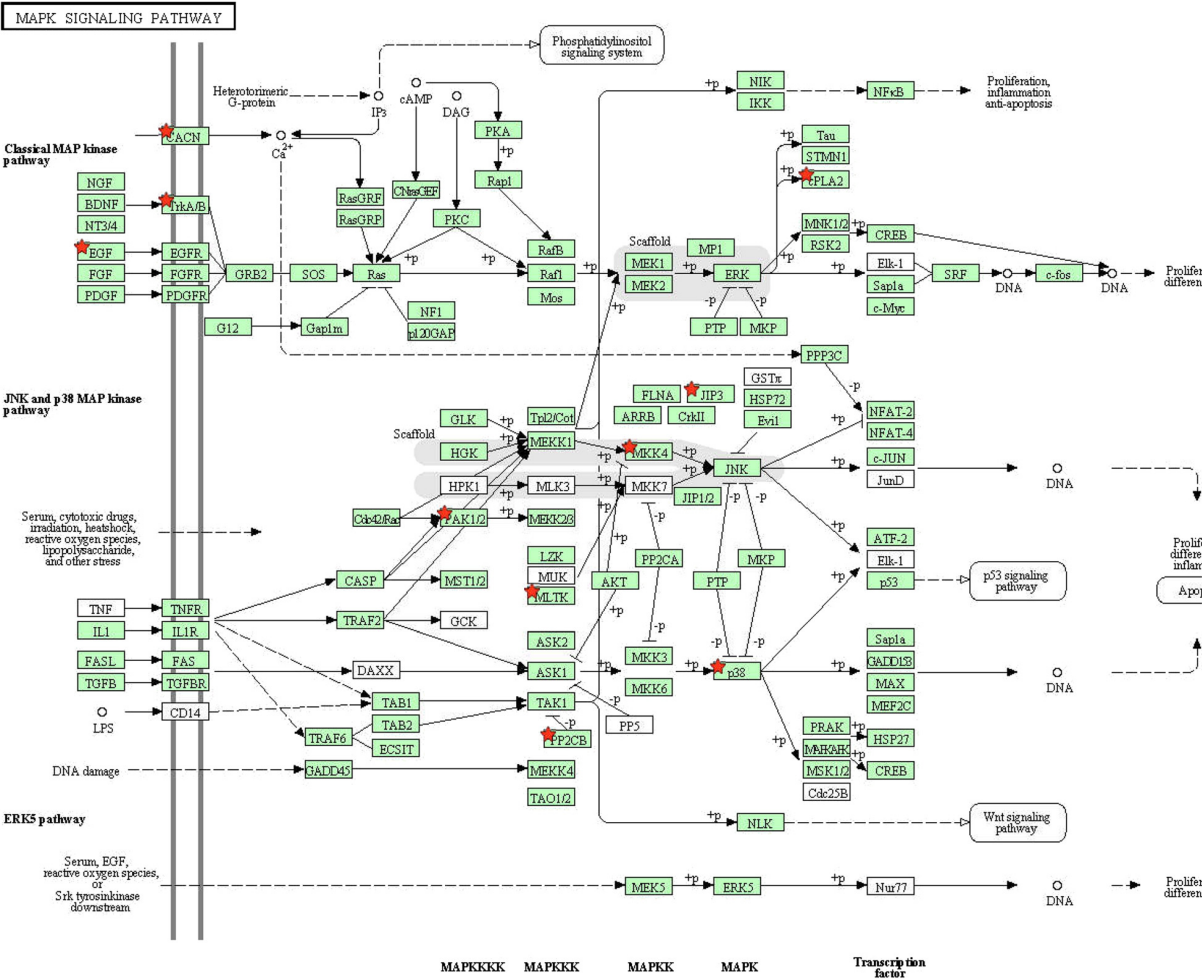
MAPK signaling pathway in CNVRs unique to the susceptible line

A total of 399 and 409 Ensembl genes were found to overlap the CNVRs fixed within resistant and susceptible line, respectively. We performed GO and KEGG pathway analyses to explore the functions of these genes. GO analysis reported 51 terms for the resistant line, 36 of which were significant (Additional file 5: Table S8). These significant terms are involved in Rab GTPase activity, synapse and calcium channel activity, etc. For the susceptible line, 34 out of the 65 reported terms were significant (Additional file 5: Table S9). The genes are enriched in the molecular functions of protein kinase activity, binding activities, and transcription regulations. Also, KEGG analysis reveals several pathways for the susceptible line, among which the MAPK signaling pathway is significantly enriched (Additional file 5: Table S9; Fig. 4).

## Discussion

MD is the only tumor disease that can be prevented by vaccination in poultry, and has been used as a model for human tumor study [1]. Understanding the genetic basis of host resistance to MD is important not only for breeding resistant chickens, but also providing clues for human studies on similar complex diseases. A variety of genetic and genomic strategies has been taken to study the mechanism of resistance to MD [4–6, 27, 32, 33] and successfully identified several candidate genes [34]. However, the causative variations remains poorly understood. CNV is increasingly recognized as an important yet not fully studied type of genetic variation. Extensive CNV discovery studies have been conducted in chicken [24, 30, 31, 35–40], but studies evaluating the association between CNVs and complex traits have been sparse [31, 40].

In this study, we hypothesized that some common CNVs within population contribute to the resistance to MD. We performed genome-wide survey of CNVs in two well-known inbred chicken lines that differ in susceptibility to MD by next generation sequencing, in order to find some CNVs associated with the resistance variation to MD. Using two distinct analytical approaches, we identified a broad range of CNVs, ranging in size from 102 bp to 543,500 bp. The results from the two analyses showed partial overlap most likely because of the differences in declaring variants as well as differences in the size range of detected CNVs (8.4 kb vs 4.6 kb, on average). It is well established that no single algorithm can capture the entire spectrum of CNVs and results from different approaches are most likely complementary to each other [16, 23, 41, 42].

Empowered by next generation sequencing, the resolution of CNVs in this study is much higher than those declared in previous studies [30, 31]. While only over one-third of previous CNVs could be validated in our studies, reasons for the relatively small overlap could be attributed to several aspects: first, not all previous CNVs were successfully converted for comparison; second, the CNV sizes from Crooijmans et al. [30] and Luo et al. [31] are very large, whereas the mean size of CNVs from both CNVnator and Pindel in this study are much smaller. Only the largest CNVs are in the same size order of magnitude with previously identified CNVs, making it not directly comparable; third, some of the CNVs are private or rare variations. We selected four CNVs for PCR experiments to investigate the reliability of our results, all of which have not been reported by any of previous studies in the same population. The results confirmed that these CNVs are true variations, demonstrating that the results are reliable.

We observed that globally and separately, most of our CNVRs were shared by more than one individual. This is in sharp contrast with our previous findings from diverse chicken breeds, where most CNVRs were found only in one sample [24, 39]. The reduced diversity can be explained by the homogeneous genetic background due to highly inbreeding. The two chicken lines have been continuously inbred for decades, with the inbreeding coefficients within lines exceeding 99 % [17]. Nearly 40 % of the identified CNVRs were shared by the two lines, indicating that these events existed in the common ancestor, since the two lines shared some sires and dams in the initial stage [17]. The lineage-specific CNVRs may represent new events after the two lines’ divergence. Over 52 % of the line-specific CNVRs were fixed or nearly fixed within the same line, indicating that the common variation contributed more than private variation to the line-specific characteristics.

### Variation on MHC

Among the potential loci responsible for the resistance to MD, the MHC is no doubt a major locus with significant influence [43, 44]. Historically, the two chicken lines were believed to share the same MHC haplotype (B_2_) as determined serologically by erythrocyte agglutination tests and graft transplantation [17, 45]. Therefore, the resistance difference between the two lines is attributed to non-MHC loci. We inspected the copy number state of the MHC loci between the two lines to evaluate whether variation of copy number in certain regions contribute to the resistance difference. As many as 20 CNVRs were observed, 8 of which could be validated by different platforms. These findings provide additional evidences that the MHC loci are highly polymorphic, even in such highly inbred lines. From the point of evolution, it is advantageous for the population to maintain a relative high degree of diversity in the MHC loci in the context of changing circumstance. However, it is difficult to associate these CNVs with the resistance to MD according to our “common variant-disease” assumption, because most of these CNVs were private, rather than population-specific. The only line-specific CNVR (CNVR4439) shared by two chickens overlapped with the *TRIM7.1* gene. *TRIM7.1* encodes a member of tripartite motif (TRIM) families which are involved in a wide range of cellular processes and are important regulators of carcinogenesis and tumor regression [46]. However, whether TRIM family plays a role in viral infection with regard to MDV remains unknown [47].

### Integration analysis

We performed pathway analysis to investigate whether genes affected by the line-specific CNVRs involved in specific pathways or biological processes. We found the genes overlapped CNVRs unique to the susceptible line, including *EGF, CACN* and *MKK4* were significantly enriched in MAPK signaling pathway. MAPK pathway is one of the most extensively studied pathways involved in tumorgenesis [48] and has proven to be a major target of Meq during tumor formation by chromatin immunoprecipitation sequencing [49] and RNA sequencing [50]. The fact that the enrichment of genes in MAPK signaling pathway was corroborated by different strategies indicates that the MAPK signaling pathway plays an important role in host resistance to MD.

### The role of CNVs in host resistance to MD

Gene expression is crucial for many biological processes, and variation in transcriptional level plays a key role in determining the phenotypic variation [51]. Recently, genome-wide association studies of resistance to MD from Cheng et al. [11] found that more than 83 % of the additive genetic variance in MD resistance was captured by the ASE SNPs, demonstrating that variation in MD resistance are probably controlled by regulation of gene expression, and most of the ASE genes are strong candidates of underlying genes. CNVs are known able to alter gene expression, and it has been shown that CNVs contributed ~18 % of the genetic variation to gene expression [52]. Thus, we examined whether our line-specific CNVs affect some of the ASE genes and found 62 (~8 %) overlapped genes. Even though the overlap is relatively small, it has strong statistical support. It can be speculated that some of these ASE genes contribute to the variation of disease resistance through a CNV manner. However, if we assume the ASE genes are underlying genes, one may wonder the reason for the relatively small amount of overlap. One explanation is that, CNVs and SNPs have complementary roles in determining the phenotypic variation, and some of these CNVs may contribute to the remaining 20 % genetic variation in MD resistance not captured by ASE SNPs, making it not directly comparable. As a complex trait, the susceptibility to MD cannot be fully explained by the variation of a few genes, but rather tens or hundreds, or even thousands of genes with small to intermediate effects. Integrating different sources of genetic variation for functional studies is a reasonable approach to better understand the genetic basis for complex traits [53].

It should be pointed out that, the two chicken lines used in this study are highly inbred and the effective population sizes of both populations are limited. Therefore, some of these line-specific (or fixed) CNVs are generated simply due to random factors, such as genetic drift during the divergence of the two lines. These CNVs are probably functionally neutral and contribute little to the resistance or susceptibility to MD. However, it is beyond the scope of our study to distinguish these CNVs from others as the aim of this study is to provide a broad picture of the CNVs in the genomes of the two parental lines. Future studies using intercross or backcross populations with greater statistical power should address this issue. Also, the candidate genes in CNVRs need more validation to confirm whether CNVs play a role in determining the resistance or susceptibility to MD.

## Conclusions

In summary, we sequenced three chickens from each of the two chicken lines with different susceptibility to MD and performed an initial screening of CNVs in the genomes of the two parental lines that potentially involved in MD resistance. A number of line-specific CNVs were identified, most of which were fixed or nearly fixed. Pathway analysis of the genes affected by fixed CNVs provides additional lines of evidence that MAPK signaling pathway may play an important role in host response to MDV infection. Integration with functional loci identified previously reveals some CNVs potentially involved in the host response to MDV infection through altering gene expression levels. Our study provides additional insights into the genetic and genomic architecture of the two chicken lines, and the CNVs, especially the line-specific CNVs are valuable resources for future association studies.

## Additional files

Additional file 1: Table S1. Summary of identified CNVs and CNVRs in the two chicken lines. For CNVs reported by CNVnator, the copy number denoted the absolute copy number estimated by CNVnator, and for those predicted by Pindel, the absolute copy number was not available and represented by ratio of the total number of uniquely mapped reads in the population supporting the CNV to that supporting the reference. Besides, Pindel can detected some not pure deletions (some fragments are inserted in the deletion region), and the inserted fragments were listed in the “Note” column. (XLSX 1591 kb) Additional file 2: Table S2-S4. Summary (Table S2) of successfully converted CNVs (Table S3) and comparison with current study (Table S4). In the first column of Table S2, “C” denotes this CNV is from Crooijmans’s et al.’s [30] study and “L” stands for “Luo et al.’s [31] study. (XLSX 72 kb) Additional file 3: Table S5. Forward and backward primers of the validated CNVRs. (XLSX 9 kb) Additional file 4: Table S6-S7. List of ASE genes used for comparison (Table S6) and Overlap between line-specific CNVRs of high frequency with ASE genes (Table S7). ASE: allele-specific expression. (XLSX 45 kb) Additional file 5: Table S8-S9. GO and KEGG pathway analysis of genes affected by resistant-specific CNVRs (Table S8) and susceptible-specific CNVRs (Table S9). (XLSX 27 kb)

## Abbreviations

ASE: Allele-specific expression
CNV: Copy number variation
CNVR: Copy number variation region
DEG: Differentially expressed gene
GO: Gene ontology
GWAS: Genome-wide association study
KEGG: Kyoto Encyclopedia of Genes and Genomes
MAPK: Mitogen-activated protein kinase
MD: Marek’s disease
MDV: Marek’s disease virus
MHC: Major histocompatibility complex
QTL: Quantitative trait loci

## References

[1] Osterrieder N, Kamil JP, Schumacher D, Tischer BK, Trapp S (2006). Marek's disease virus: from miasma to model. Nature Reviews Microbiology.

[2] Gimeno IM (2008). Marek's disease vaccines: a solution for today but a worry for tomorrow?. Vaccine.

[3] Biggs PM, Nair V (2012). The long view: 40 years of Marek's disease research and Avian Pathology. Avian Pathology: Journal of the WVPA.

[4] Bumstead N (1998). Genomic mapping of resistance to Marek's disease. Avian Pathology.

[5] Vallejo RL, Bacon LD, Liu HC, Witter RL, Groenen MA, Hillel J (1998). Genetic mapping of quantitative trait loci affecting susceptibility to Marek's disease virus induced tumors in F2 intercross chickens. Genetics.

[6] Yonash N, Bacon LD, Witter RL, Cheng HH (1999). High resolution mapping and identification of new quantitative trait loci (QTL) affecting susceptibility to Marek's disease. Animal genetics.

[7] Heifetz EM, Fulton JE, O'Sullivan NP, Arthur JA, Wang J, Dekkers JC (2007). Mapping quantitative trait loci affecting susceptibility to Marek's disease virus in a backcross population of layer chickens. Genetics.

[8] Li G, Li D, Yang N, Qu L, Hou Z, Zheng J (2014). A genome-wide association study identifies novel single nucleotide polymorphisms associated with dermal shank pigmentation in chickens. Poultry science.

[9] Cheng HH, MacEachern S, Subramaniam S, Muir WM (2012). Chicks and single-nucleotide polymorphisms: an entrée into identifying genes conferring disease resistance in chicken. Animal Production Science.

[10] Scherer SW, Lee C, Birney E, Altshuler DM, Eichler EE, Carter NP (2007). Challenges and standards in integrating surveys of structural variation. Nature genetics.

[11] Cheng HH, Perumbakkam S, Black-Pyrkosz A, Dunn JR, Muir WM. ASE screening demonstrates that variation in genetic resistance to MD in chicken is mainly controlled at the transcriptional level. In: 10th World Congress on Genetics Applied to Livestock Production. Vancouver: American Society of Animal Science (ASAS); 2014.

[12] Clop A, Vidal O, Amills M (2012). Copy number variation in the genomes of domestic animals. Animal Genetics.

[13] Hollox EJ, Hoh BP (2014). Human gene copy number variation and infectious disease. Human Genetics.

[14] Eichler EE, Flint J, Gibson G, Kong A, Leal SM, Moore JH (2010). Missing heritability and strategies for finding the underlying causes of complex disease. Nature Reviews Genetics.

[15] Stankiewicz P, Lupski JR (2010). Structural variation in the human genome and its role in disease. Annual Review of Medicine.

[16] Mills RE, Walter K, Stewart C, Handsaker RE, Chen K, Alkan C (2011). Mapping copy number variation by population-scale genome sequencing. Nature.

[17] Stone HA (1975). Use of highly inbred chickens in research.

[18] Hunt HD, Fulton JE (1998). Analysis of polymorphisms in the major expressed class I locus (B-FIV) of the chicken. Immunogenetics.

[19] Bacon LD, Hunt HD, Cheng HH (2000). A review of the development of chicken lines to resolve genes determining resistance to diseases. Poultry Science.

[20] Sambrook J, Russell DW (2001). Molecular Cloning: A Laboratory Manual.

[21] Yan Y, Yi G, Sun C, Qu L, Yang N (2014). Genome-Wide Characterization of Insertion and Deletion Variation in Chicken Using Next Generation Sequencing. PloS one.

[22] Li H. Aligning sequence reads, clone sequences and assembly contigs with BWA-MEM. arXiv preprint arXiv:13033997 2013. http://arxiv.org/pdf/1303.3997v2.pdf

[23] Abyzov A, Urban AE, Snyder M, Gerstein M (2011). CNVnator: an approach to discover, genotype, and characterize typical and atypical CNVs from family and population genome sequencing. Genome Research.

[24] Yi G, Qu L, Liu J, Yan Y, Xu G, Yang N (2014). Genome-wide patterns of copy number variation in the diversified chicken genomes using next-generation sequencing. BMC Genomics.

[25] Ye K, Schulz MH, Long Q, Apweiler R, Ning Z (2009). Pindel: a pattern growth approach to detect break points of large deletions and medium sized insertions from paired-end short reads. Bioinformatics.

[26] Huang DW, Sherman BT, Lempicki RA (2009). Systematic and integrative analysis of large gene lists using DAVID bioinformatics resources. Nature Protocols.

[27] Perumbakkam S, Muir WM, Black-Pyrkosz A, Okimoto R, Cheng HH (2013). Comparison and contrast of genes and biological pathways responding to Marek's disease virus infection using allele-specific expression and differential expression in broiler and layer chickens. BMC Genomics.

[28] R Core Team: R. A language and environment for statistical computing. R Foundation for Statistical Computing 2015. URL: http://www.R-project.org/.

[29] Lalitha S (2000). Primer premier 5. Biotech Software & Internet Report: The Computer Software Journal for Scient.

[30] Crooijmans RP, Fife MS, Fitzgerald TW, Strickland S, Cheng HH, Kaiser P (2013). Large scale variation in DNA copy number in chicken breeds. BMC Genomics.

[31] Luo J, Yu Y, Mitra A, Chang S, Zhang H, Liu G (2013). Genome-wide copy number variant analysis in inbred chickens lines with different susceptibility to Marek's disease. G3 (Bethesda, Md).

[32] Yu Y, Luo J, Mitra A, Chang S, Tian F, Zhang H (2011). Temporal transcriptome changes induced by MDV in Marek's disease-resistant and -susceptible inbred chickens. BMC Genomics.

[33] McElroy JP, Dekkers JC, Fulton JE, O'Sullivan NP, Soller M, Lipkin E (2005). Microsatellite markers associated with resistance to Marek's disease in commercial layer chickens. Poultry Science.

[34] Cheng HH, Kaiser P, Lamont SJ (2013). Integrated Genomic Approaches to Enhance Genetic Resistance in Chickens. Annual Review of Animal Biosciences.

[35] Wang X, Nahashon S, Feaster TK, Bohannon-Stewart A, Adefope N (2010). An initial map of chromosomal segmental copy number variations in the chicken. BMC Genomics.

[36] Wang Y, Gu X, Feng C, Song C, Hu X, Li N (2012). A genome-wide survey of copy number variation regions in various chicken breeds by array comparative genomic hybridization method. Animal Genetics.

[37] Jia X, Chen S, Zhou H, Li D, Liu W, Yang N (2013). Copy number variations identified in the chicken using a 60 K SNP BeadChip. Animal Genetics.

[38] Fan WL, Ng CS, Chen CF, Lu MY, Chen YH, Liu CJ (2013). Genome-wide patterns of genetic variation in two domestic chickens. Genome Biology and Evolution.

[39] Han R, Yang P, Tian Y, Wang D, Zhang Z, Wang L (2014). Identification and functional characterization of copy number variations in diverse chicken breeds. BMC Genomics.

[40] Zhang H, Du ZQ, Dong JQ, Wang HX, Shi HY, Wang N (2014). Detection of genome-wide copy number variations in two chicken lines divergently selected for abdominal fat content. BMC Genomics.

[41] Yoon S, Xuan Z, Makarov V, Ye K, Sebat J (2009). Sensitive and accurate detection of copy number variants using read depth of coverage. Genome Research.

[42] Alkan C, Coe BP, Eichler EE (2011). Genome structural variation discovery and genotyping. Nat Rev Genet.

[43] Cole RK (1968). Studies on genetic resistance to Marek's disease. Avian Diseases.

[44] Briles WE, Stone HA, Cole RK. Marek's disease: effects of B histocompatibility alloalleles in resistant and susceptible chicken lines. Science (New York, NY). 1977;195(4274):193–5. 10.1126/science.831269 831269

[45] Pazderka F, Longenecker B, Law GJ, Stone H, Ruth R (1975). Histocompatibility of chicken populations selected for resistance to Marek's disease. Immunogenetics.

[46] Hatakeyama S (2011). TRIM proteins and cancer. Nature Reviews Cancer.

[47] Shiina T, Briles WE, Goto RM, Hosomichi K, Yanagiya K, Shimizu S (2007). Extended gene map reveals tripartite motif, C-type lectin, and Ig superfamily type genes within a subregion of the chicken MHC-B affecting infectious disease. Journal of Immunology (Baltimore, Md : 1950).

[48] Dhillon AS, Hagan S, Rath O, Kolch W (2007). MAP kinase signalling pathways in cancer. Oncogene.

[49] Subramaniam S, Johnston J, Preeyanon L, Brown CT, Kung HJ, Cheng HH (2013). Integrated analyses of genome-wide DNA occupancy and expression profiling identify key genes and pathways involved in cellular transformation by a Marek's disease virus oncoprotein, Meq. Journal of Virology.

[50] Subramaniam S, Preeyanon L, Cheng HH (2013). Transcriptional profiling of mEq-dependent genes in Marek's disease resistant and susceptible inbred chicken lines. PloS one.

[51] Albert FW, Kruglyak L (2015). The role of regulatory variation in complex traits and disease. Nature Reviews Genetics.

[52] Stranger BE, Forrest MS, Dunning M, Ingle CE, Beazley C, Thorne N (2007). Relative impact of nucleotide and copy number variation on gene expression phenotypes. Science.

[53] Peterson RE, Maes HH, Lin P, Kramer JR, Hesselbrock VM, Bauer LO (2014). On the association of common and rare genetic variation influencing body mass index: a combined SNP and CNV analysis. BMC Genomics.

